# Neuroimaging and Cognitive Function in Sickle Cell Disease: A Systematic Review

**DOI:** 10.3390/children10030532

**Published:** 2023-03-09

**Authors:** Suad S. Abdi, Michelle De Haan, Fenella J. Kirkham

**Affiliations:** 1 Developmental Neurosciences Section, UCL Great Ormond Street Institute of Child Health, London WC1N 1EH, UK; 2 Clinical and Experimental Sciences, University of Southampton, Southampton SO16 6YD, UK; 3 Child Health, University Hospital Southampton, Southampton SO16 6YD, UK

**Keywords:** anemia, sickle cell, neuropsychology, intelligence quotient, transcranial Doppler, magnetic resonance imaging, connectome

## Abstract

Sickle cell disease (SCD) is the most common inherited single-gene disease. Complications include chronic anaemia, reduced oxygen-carrying capability, and cerebral vasculopathy, resulting in silent cerebral infarction, stroke, and cognitive dysfunction with impairments in measures of executive function, attention, reasoning, language, memory, and IQ. This systematic review aims to investigate the association between neuroimaging findings and cognition in children with SCD. Searches of PubMed and Embase were conducted in March 2022. Studies were included if participants were <18 years, if original data were published in English between 1960 and 2022, if any genotype of SCD was included, and if the relationship between cognition and neuroimaging was examined. Exclusion criteria included case studies, editorials, and reviews. Quality was assessed using the Critical Appraisal Skills Programme Case Control Checklist. A total of 303 articles were retrieved; 33 met the eligibility criteria. The presence of overt or silent strokes, elevated blood flow velocities, abnormal functional connectivity, and decreased fMRI activation were associated with neuropsychological deficits in children with SCD when compared to controls. There is a critical need to address the disease manifestations of SCD early, as damage appears to begin at a young age. Most studies were cross-sectional, restricting the interpretation of the directionality of relationships. Future research employing longitudinal neuroimaging and neuropsychological assessments could improve our understanding of the cumulative consequences of SCD on the developing brain.

## 1. Introduction

**Figure 1:**
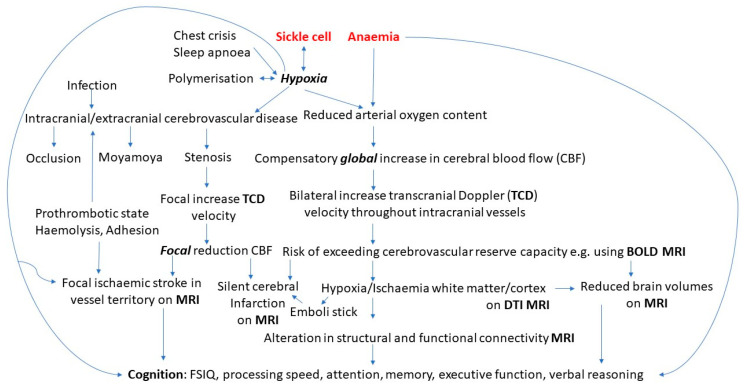
Pathophysiology of cognitive difficulties in sickle cell disease.

**Figure 2:**
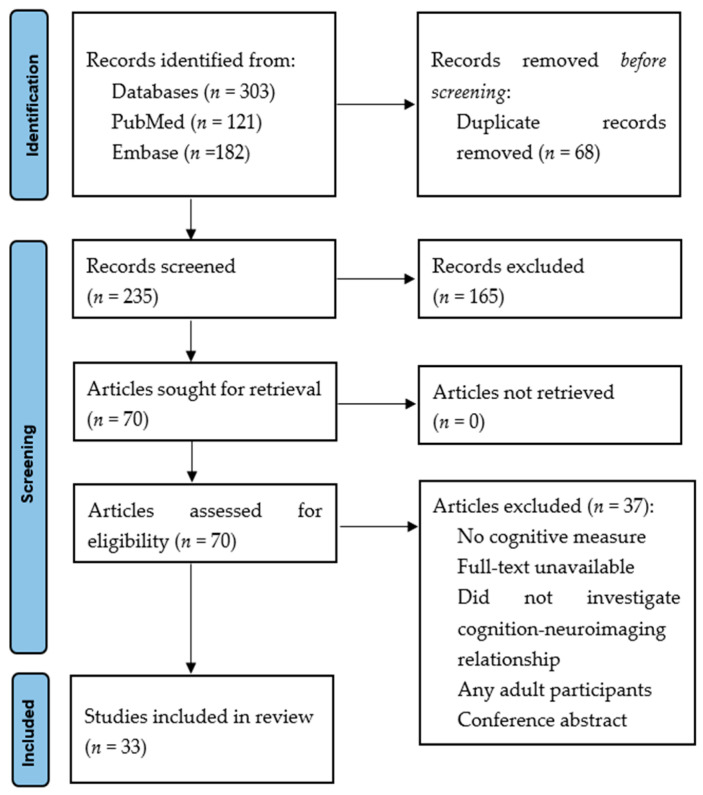
Preferred Reporting Items for Systematic Reviews and Meta-Analyses (PRISMA) flow diagram.

Sickle cell disease (SCD) refers to a group of genetic disorders characterised by misshapen red blood cells caused by the polymerisation of abnormal haemoglobin in hypoxic conditions, with approximately 300,000 babies born each year worldwide suffering from these disorders [1,2]. SCD-related complications result in anaemia, cerebrovascular disease [3], brain infarction [4], ischaemia-reperfusion injury [5,6], and increased risk of cognitive impairments [7] (Figure 1). Research has suggested most individuals with SCD experience cognitive impairments as a result of overt strokes or clinically silent cerebral infarctions (SCIs) [8]. For instance, specific impairments in writing and reading abilities have been observed in children with SCD who have experienced strokes [9], and receptive and expressive language developmental delays are frequent [10]. Studies have also shown that children with SCD and SCIs performed worse on tests of vocabulary, coordination, visual-motor speed, and arithmetic when compared to children with SCD with no imaging abnormalities [11,12]. The most common SCD genotype is haemoglobin SS (HbSS) [13]. The relationship between cerebrovascular complications and cognitive function (Figure 2) can be investigated using transcranial Doppler ultrasonography (TCD), and, in addition to structural magnetic resonance imaging (MRI), other MRI modalities can be used, including functional magnetic resonance imaging (fMRI) and diffusion magnetic resonance imaging (dMRI).

Vaso-occlusion arises when sickled red blood cells obstruct blood flow to the extent that tissues are oxygen-deprived [14]. In response, an inflammatory reaction is triggered, which may lead to the narrowing of vessels, including intracranial and extracranial arteries (Figure 2). This increases the risk of vascular nitric oxide release, endothelial activation, and adherence of red and white cells, platelets and microparticles, especially as contemporaneous reduction of protein S and C shifts the blood towards a more prothrombotic condition [15,16,17], increasing the risk of arterial, as well as venous, thrombosis [18].

An estimated 10% of children with HbSS will have an overt stroke without screening and preventative treatment [19,20]. Transcranial Doppler is used to measure the velocity in the intracranial arteries, typically the middle cerebral (MCA) but also the anterior cerebral (ACA) and distal internal carotid arteries (dICA). Abnormal ICA/MCA velocities ≥200 cm/s, secondary to narrowing of the arteries or high cerebral blood flow, are associated with a 40% risk of stroke over the subsequent 3 years, while conditional velocities (170–199 cm/s) predict a 7% risk. Regular blood transfusion or hydroxyurea treatment for those with abnormal velocities reduces the stroke risk substantially. Cognitive difficulties may also be related to high cerebral blood flow velocities.

Neuroimaging plays a crucial role in the screening, diagnosis, treatment, and prevention of brain damage in children with SCD, as around one-third sustain SCIs in childhood [19,20], with more than half affected by young adulthood [21]. SCIs may go undiscovered, but they can have life-altering implications, as there is evidence for association with reduced global intellectual functioning [22], poor educational performance [22], and decreased quality of life [23], as well as potentially impeding the normal development of brain structure and function. There have been several systematic reviews that have examined the neurological correlates of the cognitive deficits identified in individuals with SCD [24,25,26]. Kawadler et al. found that children with SCD and SCI had lower IQ scores than children with SCD with no SCI [25], but those with no MRI abnormalities displayed lower IQs than healthy participants, suggesting that other factors (biological, environmental, and socio-economic) play a more substantial role in cognitive function [25]. Similarly, a previous meta-analysis concluded that children with SCD without MRI abnormalities were at risk of cognitive difficulties [24]. Prussien et al. found participants with SCD, including those without SCI or overt strokes, showed significant impairments in attention, FSIQ, executive function, and verbal reasoning [26], positing that anaemia and altered cerebral haemodynamics [27] impact neurological functioning [28].

MRI is a reliable, safe, and practical technique for the detection and management of SCD-related neurological damage [29]. In addition to the detection of SCI, quantitative MRI can be used to examine volume reduction in white matter (WM) [30,31], deep grey matter [32] and the cortex [33], which has, in SCD, been associated with impaired cognition, even in the absence of focal brain injuries. Additionally, arterial vasculopathy may induces persistent focal ischaemia in the brain tissue of individuals with SCD, which results in microstructural alterations such as increased intercellular water content and WM fibre degradation [34]. Diffusion-weighted imaging, which detects acute ischaemia, has reported damage to WM tract integrity and density in SCD, which is related to cognitive deficits [35]. There has not yet been a systematic review examining the relationship between quantitative neuroimaging modalities and cognitive function in children with SCD.

fMRI can be used to assess blood oxygen level-dependent (BOLD) signals in distinct brain areas during resting states or task performance in SCD [36]. The BOLD signal changes in the brain are temporally associated across functionally related regions [37,38]. However, aberrant cerebral haemodynamics and anaemia may make it challenging to detect fMRI activation in patients with SCD [39,40], even if the brain activity itself is undisturbed by the disease. Studies of patients with SCD have demonstrated that abnormal resting cerebral blood flow (CBF) reduces the BOLD response due to a diminished capability to increase blood flow in response to an increase in brain activity [41].

Given the importance of cerebrovascular disease and brain structure and function in cognition and the contributing role of neuroimaging in diagnosing and identifying those at greater risk for complications in SCD, the purpose of this review was to examine how quantitative imaging modalities relate to cognition in children with SCD, identify measures that relate to neuropsychological functioning, and ascertain key regions for future studies. Further insight into the pathophysiology of SCD will also allow for the implementation of different targets for intervention (Figure 1).

## 2. Materials and Methods

### 2.1. Search Strategy and Selection Criteria

This review synthesises research on neuroimaging and cognition in paediatric patients with SCD using electronic searches of the PubMed and Embase databases conducted in March 2022. Table 1 illustrates the search strategy that was employed in one of the databases. The Preferred Reporting Items for Systematic Reviews and Meta-Analyses (PRISMA) guidelines were followed for this review [42].

Inclusion and exclusion criteria for articles were created and are summarised in Table 2. References of excluded articles were examined to identify any appropriate articles for inclusion. The search was conducted from 1960 to 2022 to provide a complete and exhaustive list of the neuroimaging research in paediatric SCD. No limits were applied for foreign articles, but studies were only included if they had been translated into English due to time constraints.

EndNote X9 (The EndNote Team, 2013) was used to store results, with all duplicates omitted. One reviewer examined titles and abstracts, and articles were retained if they collected neuroimaging data and cognitive performance in SCD. The remaining papers’ full texts were obtained, and eligibility was assessed using inclusion and exclusion criteria (Table 2).

### 2.2. Quality Assessment

To assess quality, the Critical Appraisal Skills Programme Case Control Checklist was used. Articles were evaluated, which included questions on the suitability of controls (data from normative databases, community/sibling control) and the validity of neuropsychological assessments and neuroimaging used in articles of interest. The checklist included 3 categories with quality scores of 0 (No) or 1 (Yes), from which a total score was calculated. Articles were classified into good quality (66% or higher), satisfactory quality (36–65%), and poor quality (0–35%).

## 3. Results

The search retrieved 303 articles. A total of 68 duplicates were removed, and 235 articles were assessed by title and abstract. A total of 165 articles were excluded as they did not meet the eligibility criteria. Full texts of 70 articles were analysed, of which 33 met the inclusion criteria. The PRISMA flow diagram (Figure 2) outlines the specific selection process.

### 3.1. Data Extraction

The methodological characteristics of included studies presented by imaging modality are summarised in Table 3 (A), (B), (C), (D) and (E).

Quality appraisal scores for each paper are summarised in Table 4. Thirteen articles (39.3%) were of good quality, and 20 articles (60.6%) were of satisfactory quality.

### 3.2. Characteristics of Articles in Review

Most studies used cross-sectional designs (*n* = 29; 87.8%), with two longitudinal studies and two retrospective cohort studies. Publication years ranged from 1993 to 2022, with seven (21.2%) from the 1990s, fourteen (42.4%) from the 2000s and twelve (36.3%) from 2011–2022. Articles’ sample sizes varied from 14 to 373, with mean ages spanning 3 months to 16.34 years. All SCD genotypes were included, with HbSS represented in all studies, sickle beta-thalassaemia in 14 (42.4%), and HbSC in 12 (36.3%). Of the 33 studies included, 23 (69.6%) included SCI+ and/or stroke groups, and 13 (39.3%) included healthy controls. Most studies were conducted in the USA (*n* = 21; 63.6%), with 3 studies (9.1%) in Italy, 2 studies (6.1%) in the Netherlands, 3 studies (9.1%) in the UK, 1 study (3%) in Canada, 1 study (3%) in Tanzania, 1 study (3%) in Nigeria, and 1 study (3%) in France.

### 3.3. Study Outcomes

A. TCD

CBFV measured using TCD was investigated in 11 (33.3%) studies of infants, toddlers, preschoolers, and school-age children and adolescents with SCD (Table 3 (A)). Hogan et al. [43] and Schatz et al. [44] reported that in 9-month-old infants with SCD and in 26-month-old children with SCD, high CBF velocities were related to a moderate to high risk of developmental delay when compared to controls. Although narrowing of the intracranial arteries plays a role, the increase in CBF velocity is mainly related to the anaemia and may affect brain development over a longer period. However, Aygun et al. [45] found no relationship between TCD velocities and scores on an academic screening measure in children with SCD (mean age of 3.5 years). Differing family and environmental factors may partially explain these contradictory TCD findings.

Sanchez et al. [46] reported that higher TCD velocities were negatively related to phonological processing and syntactical ability in children with HbSS genotypes of SCD. Kral et al. [49] found that children with abnormal velocities showed greater verbal intelligence impairments than those with lower conditional TCD values. Conversely, Kral et al. [50] reported most subjects (*n* = 22) displayed elevated TCD velocities and found a positive relationship between better verbal memory and raised TCD values. The abnormal TCD group’s performance in both studies may have been influenced by the chronic blood transfusions patients underwent. Prussien et al. [52] found participants not receiving chronic transfusion with higher TCD velocity had poorer performance on tests of executive function and perceptual reasoning.

Onofri et al. [51] and Strouse et al. [53] included younger participants (mean age 8–9 years) and did not find any relationship between cognition and TCD velocities. Similarly, Hijmans et al. [48] found no correlation between neuropsychological measures (including sustained attention, IQ, and inhibition) and TCD values. Hijmans et al. reported children with asymmetries in CBF scored higher on 8 of 13 neuropsychological assessments than those without asymmetries. The researchers found only one test of sustained attention statistically significant; children with right-left asymmetries had lower mean reaction times than those without asymmetries [48]. Lastly, Bernaudin et al. [47] found that elevated TCD measures were related to poorer neuropsychological outcomes and lower IQ scores in children with SCD. However, there were no significant differences between the children with abnormal or normal TCD values after the patients with stroke were excluded.

B. Structural MRI: infarction

Cognitive impairments were reported in children with SCD using validated assessments of global intellectual function (intelligence quotient (IQ)) and academic achievement. Twenty-one studies (63.6%) assessed brain structure (Table 3 (B)), specifically SCI presence, with most including number and volume as well. Fourteen (66.6%) MRI studies found children with SCD and overt stroke or SCI performed more poorly on neuropsychological tests, including full-scale IQ and measures of executive function, visuomotor skills, mathematical ability, working memory, verbal IQ, and attention, than children with SCD and normal-appearing MRI [11,12,28,47,54,55,56,60,61,62,64,66,68].

Seven (33.3%) studies did not find a significant association between MRI abnormalities and impaired cognition [48,51,57,58,59,63,65] (Table 3 (B)). Grueneich et al. [57] suggested these null findings were related to MRI abnormalities being associated with greater variability in cognition. The authors posited that children with pathopsychological changes develop a pattern of strengthening certain cognitive abilities to accommodate for weak abilities that have been negatively impacted by these changes. This results in uneven neuropsychological profiles while maintaining an overall level of functioning within normal limits on assessments [57]. Hijmans et al. [48] and Montanaro et al. [59] found there were no significant differences in neuropsychological function and intelligence between children with SCI on MRI and children with normal-appearing MRI. The discrepancy between these results and previous studies may be due to confounding variables (e.g., socio-cultural) as well as technical MRI differences. Onofri et al. [51] found no relationship between impaired IQ and SCI, despite 40% of the patients presenting with SCI on MRI. However, participants were younger than in the other studies.

C. Structural MRI: volume (Table 3 (C))

Chen et al. [67] reported that, compared to their counterparts in the high-IQ group, children with SCD in the low-IQ group had reduced grey matter volume in the frontal, parietal, and temporal lobes (Table 3 (C)). Schatz and Buzan [68] found that the size of the CC was associated with working memory, speed of production, and distractibility.

D. Structural MRI: diffusion tensor imaging for microstructure (Table 3 (D))

Scantlebury et al. [69] found the structural integrity of WM pathways was affected in children with SCD and reported increased apparent diffusion coefficient values in several brain areas associated with deficits in processing speed and working memory. Stotesbury et al. [70] found widespread WM anomalies were significantly correlated with slower processing speed, even when there was no evidence of infarct on MRI.

E. Functional MRI for connectivity (Table 3 (E)).

Colombatti et al. [71] reported that patients with SCD exhibited greater connectivity in the precuneus than controls, which was pronounced in children with poorer neuropsychological functioning. The connectivity within the default-mode network may be reduced in patients with severe cognitive deficits, as it is involved in speculative processes such as planning and memory. Zou et al. [41] found children with SCD had diminished BOLD responses within the visual cortex during black-and-white visual stimulation tests compared to non-SCD children with posterior fossa tumours, which was associated with lower scores on a test of IQ [41].

## 4. Discussion

This systematic review found that children with SCD showed poorer cognition when compared to controls. The following characteristics related to poorer neuropsychological functioning in children with SCD: elevated blood flow velocities, presence of overt stroke or SCI, decreased fMRI activation, and abnormal functional connectivity. The consistent finding of neuropsychological deficits is notable considering the variability across articles in neuroimaging modalities, patient criteria for inclusion, and cognitive assessments. The discrepant findings reported by some MRI studies [57] might be explained by differences in their study samples and methodology. For example, some studies excluded participants based on chronic transfusion [48], others had small samples [51], and some included mixed immigrant cohorts from various socio-economic backgrounds including bilingual or multilingual speakers [48,59], making generalisation more difficult.

Findings indicated that haemoglobin may be a marker for decreased oxygen delivery and that reduced oxygen saturation in children with SCD may indicate cerebral hypoxia, resulting in neuropsychological deficits. Hijmans et al. [48] reported haemoglobin was a significant predictor of verbal memory and that anaemia was a greater predictor of neuropsychological impairments than SCI on MRI. Similarly, Steen et al. [28] reported that severity of chronic anaemia accounted for 23% of the variance in full-scale IQ in children with SCD without strokes.

A review of the research on cognition and TCD velocities in SCD also revealed mixed findings. For example, Kral et al. [50] found that children with SCD with the highest TCD velocities receiving chronic blood transfusions had better verbal memory than those with moderately high velocities who remained untreated. These results imply that blood transfusions may affect cognition by improving oxygen saturation and haemoglobin. Conversely, some TCD studies had null findings but provided limited information related to potential confounding variables (e.g., medications and blood transfusion), which limits the ability to interpret neuropsychological results [45,48,51,53].

Research has also shown children with SCD have higher resting CBF velocity than controls [72]. Therefore, as CBF velocities in these patients may be at maximum during resting states, the need for increased CBF during executive function and working memory processes in the prefrontal cortex may go unfulfilled [52]. According to interactive specialisation theory, executive function development requires interconnectivity between multiple brain areas [73]. This theory may be especially relevant to SCD. Even in the absence of stroke, blood flow and WM integrity are impaired in children. WM pathways and sufficient blood flow are critical for integrating information between different brain areas in circuits connected to executive functioning [70,73,74]. Increasing global CBF may be a risk factor and a response to cerebral hypoxia; however, these compensatory strategies may be inadequate when there is a further demand for an increase in CBF, raising the risk of cognitive decline.

Few studies combine resting-state fMRI with task-based results, partly because the assumptions used to calculate the BOLD response may not be accurate in anaemia; despite this, differences between children may still be clinically significant. Zou et al. [41] reported that children with SCD had reduced visual cortical (V1) activity, which is associated with lower IQ [41]. Because of the small sample, these results must be carefully interpreted; however, patients with SCD not receiving a disease-modifying treatment exhibited no V1 activity under the same stimuli and poorer IQ scores, suggesting an association between neuropsychological deficits and untreated SCD.

Increases in the mean diffusivity of several brain areas, indicating damage to myelin integrity and neural microstructures, are correlated with poor processing speed in patients with SCD [69,70]. Recently, Chai et al. [74] speculated that the maintenance of CBF to the GM is prioritised over WM to maintain the health of neurons essential for survival, as infarcts in GM are immediately devastating. In contrast, infarcts in the WM are ‘silent’ because they merely inhibit fast information processing. However, WM strokes can substantially hinder crucial aspects of the day-to-day lives of patients with SCD, even if they do not result in major motor impairments.

### 4.1. Methodological Issues and Future Directions

There are several methodological issues in the SCD literature. First, apart from two longitudinal studies, all articles investigating neuroimaging and cognition were cross-sectional, limiting any interpretation of the directionality of relationships. Moreover, because of the challenges of determining when an SCI has occurred, few studies can explore the role of time since cerebrovascular injury is a potential mediating variable related to neuropsychological impairments in children. Studies including neuroimaging rarely assess other possible contributors such as sleep apnoea and numerous school absences on cognition. Research shows environmental factors, in addition to biological risks, are significantly related to executive function and IQ in SCD [22]. Children with SCD frequently grow up in low socio-economic status households, and factors including home environment, parental education, and family income are associated with neuropsychological functioning [22]. More studies with larger sample sizes are required to evaluate the mechanisms and risk factors for neuropsychological deficits in SCD in the presence and absence of overt stroke and SCI. For these reasons, it is crucial to differentiate between socio-economic and psychological aspects, the indirect impact of chronic illness, and the disease-related effect on brain function [75]. This necessitates the use of both neuropsychological studies and longitudinal neuroimaging, as well as clinical and demographic data.

### 4.2. Clinical Implications

This review aims to raise awareness of the need for more research focusing on the mediating role of environmental/psychosocial factors in the relationship between neuroimaging and neuropsychological deficits in SCD. In clinical settings, objective, standardised assessments should be given to children, caregivers, and teachers to ensure multiple sources of information are collected, followed by carefully formulating targeted interventions for children with SCD and their families. This information is critical in identifying vulnerable children with SCD and families who require additional support. The paediatric neurocognitive interventions model is a comprehensive framework that could aid in the development of appropriate treatments for children with SCD [76]. The model emphasises the importance of a developmental, multi-dimensional treatment that focuses on behaviour, emotions, and cognition. The foundation of this approach involves tackling the psychosocial and systemic requirements of children. Therapies are individually tailored to identify neuropsychological impairments, progressing from externally supported compensatory tactics to self-directed compensatory approaches. In children with SCD, there is a critical need for holistic treatments that address foundational needs that are not yet met due to living with a chronic illness (e.g., mental health, social isolation, hospitalisations), as well as targeted interventions for cognitive deficits.

A significant problem within this patient group is that decreased cognitive functioning may have a negative impact on medication adherence, self-care, and clinical attendance, particularly as children with SCD get older [77], with executive function deficits predicting hydroxyurea non-adherence [78]; clinician monitoring of adherence is, therefore, essential [79]. Moreover, recall barriers (forgetfulness) were identified by Fogarty et al. [80] as the biggest challenge of medication adherence for Irish teenagers, who are, however, amenable to the use of mobile apps to monitor, be notified of, or obtain information on SCD treatments [80].

### 4.3. Limitations

There are some limitations to this review. Non-English language published studies were not reviewed, and only two databases were searched. One reviewer carried out the review and quality appraisal. As with other reviews of retrospectively published findings, the results may be biased as significant correlations are more likely to be reported than non-significant relationships [81]. Another limitation of this review is the exclusion of other MRI measures, including cerebral blood flow and oxygen extraction fraction (OEF), and their relationship with cognitive function, recently reviewed by Ramos et al. [27]. Lastly, although articles used standardised neuropsychological assessments, they differed significantly, making direct comparisons challenging. Therefore, it was difficult to determine how certain neuroimaging findings were differentially related to neuropsychological domains.

## 5. Conclusions

This review highlights the prevalence of cerebrovascular disease in children with SCD and finds that more research is needed as neuroimaging findings do not fully explain cognitive deficits. Results suggest macro-and microscopic brain damage occurring due to insufficient blood supply and energy-intensive processes, with decreased blood flow leading to atrophy of cortical areas and neuronal cell death. This review also emphasises the need to address the disease manifestations of SCD at a young age. Finally, this review indicates there is great promise in utilising neuroradiological markers for early risk stratification so that children with SCD at the highest risk for cognitive deficits can be targeted early for interventions. With successful early risk stratification, interventions and treatments can also be tailored to specific patterns of strengths and weaknesses.

## Figures and Tables

**Table 1:** Search strategy in PubMed.

Concept	MeSH	Key Terms
Sickle Cell Disease	Hemoglobin, Sickle, Anemia, Sickle Cell, Hemoglobin SC Disease, Haemoglobin, Sickle, Anaemia, Sickle Cell, Haemoglobin SC Disease	“sickle” OR “sickle cell”
Advanced Neuroimaging	Diffusion Tensor Imaging, Magnetic Resonance Imaging, Ultrasonography, Doppler, Transcranial	“magnetic resonance imaging” OR “MRI” OR “transcranial Doppler” OR “diffusion weighted MRI” OR “diffusion magnetic resonance imaging” OR “DTI” OR “functional MRI” OR “connectome” OR “fMRI” OR “diffusion tensor tractography” OR “functional neuroimaging” OR “brain mapping” OR “brain volume” OR “functional connectivity”
Cognition	Neuropsychological Tests, Cognition, Neuropsychology, Learning, Memory, Attention, Intelligence, Executive Function	“cognition” OR “neuropsychological functioning” OR “neuropsychology” OR “learning” OR “memory” OR “attention” OR “intelligence” OR “IQ” OR “executive function *” OR “neuropsychology *” OR “cogniti *”

Note: MeSH (Medical Subject Headings) terms are controlled vocabularies produced by the National Library of Medicine used to index articles to the most specific headings in PubMed. * expansion of term.

**Table 2:** Inclusion and exclusion criteria.

Inclusion Criteria	Exclusion Criteria
Types of studies: Participants were under the age of 18 Studies published between 1960 and 2022 All the genotypes of SCD (e.g., HbSS, HbSC, HbSβ+-thalassaemia and HbSβ0-thalassaemia) Studies published original data Studies in other languages (translated) or countries	Types of studies: Systematic and other reviews Studies without original data Case studies Editorials
Methodological aspects: Studies investigated TCD, MRI, fMRI, or dMRI and its association with cognition Used a reliable and valid performance-based measure of cognitive abilities	Methodological aspects: Studies did not analyse the relationship between neuroimaging information and cognitive performance Non-paediatric participants

Abbreviations: functional magnetic resonance imaging (fMRI), magnetic resonance imaging (MRI), transcranial Doppler ultrasound (TCD), diffusion magnetic resonance imaging (dMRI).

**Table 3:** **(A).** Characteristics of transcranial Doppler (TCD) articles included in the systematic review. **(B).** Characteristics of SCI on Structural MRI articles included in the systematic review. **(C).** Characteristics of volume on structural MRI articles included in the systematic review. **(D).** Characteristics of diffusion tensor imaging articles included in the systematic review. **(E).** Characteristics of functional MRI articles included in the systematic review.

**(A)**								
**Study**	**Country**	**Study Design**	**Patient Sample: Size, Sex (%), Mean Age**	**Sample Disease** **Characteristics**	**Control Sample**	**Neuropsychological Measures**		**Main Findings**
Hogan et al., 2006 [43]	United Kingdom	Longitudinal study	Infancy; *n* = 14, 21% female, 3, 9, 12 m	100% had HbSS	*n* = 14, 58% female, ages 3, 9 and 12 months	BINS measured at 3, 9 and 12 months.		TCD velocities 68–154 cm/s Association between a greater risk of developmental delay at the age of 9 months and increased TCD velocities.
Schatz et al., 2008 [44]	United States of America	Cross-sectional design	Toddler; *n* = 50, 44% female, 2 years 26 months	62% HbSS 18% HbSC 18% HbSβ + thalassaemia2% HbSβ° thalassaemia	None	DDST-II (infant and childhood development)		Children with higher TCD velocities showed evidence of developmental delay on Denver II screening test.
Aygun et al., 2011 [45]	United States of America	Cross-sectional design	Preschool; *n* = 88, 49% female 3.5 years	50% HbSS 26% HbSC, 16% HbSβ + thalassaemia7% HbSβ° thalassaemia, 1% HbS/Hope	None (25% National Failure rate on Brigance)	BPS-II		TCD conditional 16%; abnormal 4% 44 (50%) children with SCD had Brigance scores < normal cut-off No relationship between TCD measures and academic screening tests. Psychosocial effects more profound effect on early childhood development?
Sanchez et al., 2010 [46]	United States of America	Cross-sectional design	Young school age; 5–8 years *n* = 39, 59% female, 6 years	92% had HbSS 8% HbSβ° thalassaemia	None	TOLD-P:3; Beery-VMI (visuo-motor function); WJ-III.		TCD conditional 10%; abnormal 5% Association between impaired language, specifically syntactical and possibly phonological processing, and elevated TCD velocities, but no effect on semantics or language or attention.
Bernaudin et al., 2000 [47]	France	Cross-sectional design	School age; 5–15 years *n* = 173, 49% female, 10.2 year Normal MRI (*n* = 104), Silent infarct (*n* = 17), Stroke (*n* = 11)	89.6% had HbSS 4.6% HbSβ° thalassaemia 1.7% HbSβ + thalassaemia 4.1% HbSC	*n* = 76 siblings; age range, 5–15 years	WISC-III and WIPPSI-R (IQ)		TCD conditional 9%; abnormal 9%Relationship between lower IQ scores (performance IQ and picture arrangement) and abnormal TCD. However, there was no significant difference observed after the participants with abnormal TCD and history of stroke (*n* = 5) were excluded. Neuropsychological deficits were found in both the overt (FSIQ and Performance IQ) and silent (Verbal Comprehension, Similarities and Vocabulary) stroke groups.
Hijmans et al., 2011 [48]	The Netherlands	Cross-sectional design	School age; 5–15 years, *n* = 34, 47% female, 11.8 years	88% had HbSS, 12% HbSβ° thalassaemia	None	WAIS-III/WISC-III (IQ); Stop Task (sustained attention and response inhibition); TOL planning; N-back task (working memory); Digit Span forwards and digit span backwards (verbal memory); Beery-VMI (visuo-motor function)		TCD normal 94%, conditional 6% Found no relationship between several neuropsychological measures and TCD velocities. Found no difference between participants with normal MRI and participants with silent infarcts.
Kral et al., 2003 [49]	United States of America	Cross-sectional design	*n* = 60, 57% female, 121 months	100% had HbSS	None	WASI (IQ); TMT (visual attention); CMS (working memory); DTVMI (visuo-motor integration); CPT-II (sustained attention)		TCD conditional 25%, abnormal 33% Children with abnormal TCD had lower verbal IQ, worse executive function and auditory working memory compared to conditional TCD. Children with conditional TCD scored more poorly on tests of sustained attention and executive function than those with normal TCD velocities.
Kral et al., 2006 [50]	United States of America	Cross-sectional design	School age; *n* = 27, 56% female, 10.8 years	100% had HbSS	None	WASI (IQ); TMT (visual attention); CMS (working memory); DTVMI (visuo-motor integration); CPT-II (sustained attention)		TCD conditional 33%, abnormal 48% Positive association between performance on verbal memory tests (CMS Stories) and abnormal TCD velocities: 13% variance after accounting for age and haematocrit
Onofri et al., 2012 [51]	Italy	Cross-sectional design	School age; *n* = 35, 51% female, 8.6 years	100% had HbSS	None	WPSSI and WISC-III (IQ)		TCD: conditional/abnormal 23% No association between impaired intellectual functioning and silent infarcts/TCD velocities.
Prussien et al., 2019 [52]	Nigeria	Cross-sectional design	School age; 6–13 years, *n* = 83, 55% female, 9.10 years	98.8% had HbSS 1.2% HbSβ° thalassaemia	None	Raven’s Progressive Matrices (perceptual reasoning); WISC-IV, Digit Span (working memory); TOL (executive planning)		TCD conditional 8%, abnormal 7% Found a relationship between elevated TCD velocity and impaired performance on executive function tasks—executive planning, problem-solving and auditory working memory, with the latter specifically in males.
Strouse et al., 2006 [53]	United States of America	Cross-sectional design	School age 6–12 years, *n* = 24, sex N/A, 9 years	100% had HbSS or HbSβ° thalassaemia	None	WASI (IQ)		TCD conditional 3% No significant association between intellectual function and TCD measures.
**(B)**								
**Study**	**Country**		**Study Design**	**Patient Sample: Size, Sex (%), Mean Age**	**Sample Disease Characteristics**	**Control Sample**	**Neuropsychological Measures**	**Main Findings**
Armstrong et al., 1996 [11]	United States of America		Cross-sectional design	*n* = 194, sex N/A, 6–12 years Normal MRI: (*n* = 105) Silent Infarct, (*n* = 21), Clinical History of Stroke, (*n* = 9)	70% had HbSS 30% had HbSC	None	WISC-R (IQ); WJ-R (academic achievement); Purdue Pegboard (motor speed and coordination)	Children with SCI+ performed significantly worse on several neuropsychological tests, including tests of mathematical ability, reading, visuomotor skills and IQ, compared with children with a normal MRI.
Bernaudin et al., 2000 [47]	France		Cross-sectional design	*n* = 173, 49% female, 10.2 years Normal MRI, *n* = 104; Silent Infarct, *n* = 17; Stroke, *n* = 11	89.6% had HbSS 4.6% HbSβ° thalassaemia 1.7% HbSβ + thalassaemia 4.1% HbSC	*n* = 76 siblings, age range, 5–15 years	WISC-III and WIPPSI-R (IQ)	Neuropsychological deficits were found in both the overt (FSIQ and Performance IQ) and silent (Verbal Comprehension, Similarities and Vocabulary) stroke groups.
Brown et al., 2000 [54]	United States of America		Cross-sectional design	*n* = 63, 39.7% female, 9.75 years Normal MRI, *n* = 30 Silent Infarct, *n*= 11 Stroke, *n* = 22	76.2% had HbSS 23.8% HbSC	None	WISC-III (IQ); WJ-R (academic achievement); Cancellation A’s Task (sustained attention); Trail Making Test (mental flexibility, sequencing, visual search, and attention); Boston Naming Test (expressive language); RAN (reading ability); Purdue Pegboard (motor speed and coordination)	Found that children with silent and overt strokes scored lower on tests of executive function and sustained attention when compared to children with normal MRI scans. There were no statistically significant differences on tests of academic achievement, visuomotor skills, or global intellectual function; however, participants with overt stroke did perform worse than children with silent infarcts and normal MRI.
Craft et al., 1993 [55]	United States of America		Cross-sectional design	*n* = 29, Normal MRI, *n* = 12, 66.6% female, 10.3 years Stroke, *n* = 17 Anterior infarctions, *n* = 6, 50% female, 10.8 years Diffuse infarctions, *n* = 11, 54.5% female, 10.9 years	100% had HbSS	*n* = 20 siblings of participants with SCD, 70% female, 11.2 years	WISC-R; WJ-R; CAVLT	Participants with diffuse cortical strokes demonstrated reduced spatial function, while participants with anterior cerebral infarctions had deficits in attention (more intrusion errors). On memory, verbal or motor tests, there were no significant differences between participants with stroke and sibling controls. Six participants had MRI signs of a stroke despite having no prior history of a neurologic injury. Similar to that of participants with overt stroke, these children’s neuropsychological performance was impaired when compared to that of sibling controls. Participants with SCD but no evidence of stroke were no different from sibling controls.
Gold et al., 2008 [56]	United States of America		Retrospective study	*n* = 65, 40% female, 13 years	80% had HbSS 15% HbSC 5% HbSβ – thalassaemia	None	WISC-R or WISC-III	Found an association between the presence of silent or overt stroke in patients and low cognitive scores.
Grueneich et al., 2004 [57]	United States of America		Cross-sectional design	*n* = 31, 61% female, 11.9 years	48% had HbSS, 29% HbSC, 19% HbSβ + thalassaemia 3% HbSβ° thalassaemia	Peers	WISC-R; WRAML; Beery-VMI	No significant relationship was found between level of neuropsychological performance and MRI abnormalities. There was a relationship between increased variability in performance on assessments and imaging anomalies (i.e., perfusion anomalies in five cases, structural brain anomalies in two cases and both perfusion and structural anomalies in three cases).
Jacob et al., 2022 [58]	Tanzania		Cross-sectional design	*n* = 73, 44% female, 11.9 years	100% had HbSS	*n* = 71, 41% female, 11.1 years	WISC-IV, Coding and Symbol Search (processing speed), Block Design, Picture Concepts and Matrix Reasoning (perceptual reasoning), Digit Span and Letter-Number Sequence (working memory); Raven’s Standard Progressive Matrices (perceptual reasoning)	Found there was no significant difference on neuropsychological tests between children with and without silent infarcts or vasculopathy; however, patients with these pathologies had lower scores on certain subtests. Compared to non-SCA sibling controls, children with SCA performed worse on tests of processing speed, working memory and perceptual reasoning.
Montanaro et al., 2013 [59]	Italy		Cross-sectional design	*n* = 68, 53% female, 8.95 years	79% had HbSS, 4% HbS/β thalassaemia 16% HbS/HbC	None	WPSSI and WISC-III (IQ)	Found no relationship between intellectual functioning and silent infarcts.
Onofri et al., 2012 [51]	Italy		Cross-sectional design	*n* = 35, 51% female, 8.6 years	100% had HbSS	None	WPSSI and WISC-III (IQ)	Found no association between impaired intellectual functioning and silent infarcts.
Schatz et al., 1999 [60]	United States of America		Cross-sectional design	*n* = 28, sex not reported, 12.8 years	100% had HbSS	*n* = 17 siblings without SCD, 12.2 years	T.O.V.A; Tower of Hanoi; WCST; Intrusion Errors on CVLT-C; Perseverations on CVLT-C; Pattern Construction; Judgment of Line Orientation; Position Discrimination; Visual Form Discrimination; Shape Discrimination; Visual Closure; Word Definitions; Peabody Picture Vocabulary Test; Picture Vocabulary; Word Fluency	Found that executive abilities and attention were impaired in participants with anterior cerebral infarctions, while spatial abilities were impaired in participants with more widespread cerebral infarctions. The size of a cerebral infarct was related to language and spatial performance but not to performance in other neuropsychological domains (memory, executive function, and attention).
Schatz et al., 2002 [61]	United States of America		Cross-sectional design	*n* = 27 Lesion: (*n* = 18, 39% female, 12.4 years) No lesion: (*n* = 9, 44% female, 11.6 years)	100% had HbSS	None	Wechsler Scales (Full-Scale IQ)	Children with large silent infarcts scored lower on tests of IQ compared to children with smaller lesions and those with no lesions.
Steen et al., 1998 [62]	United States of Kingdom		Cross-sectional design	*n* = 30, 46.6% female, 10.4 years Normal cMRI (*n* = 17) Abnormal cMRI (*n* = 13)	100% HbSS	*n* = 24 healthy children of African-American hospital employees, 41.6% female, 10.5 years	WISC-III or WISC-R (IQ)	Found that children with SCD without anomalies on standard MRI still had minor cellular abnormalities. Children with SCD with and without MRI anomalies performed worse on tests of FSIQ compared to race- and age-matched controls. Furthermore, children with SCD and abnormal MRI had greater impairments than children with SCD who did not have neuroimaging abnormalities. Found quantitative MRI T1 was lower than in healthy controls in caudate and cortical grey matter, as well as the ratio of grey-to-white matter.
Steen et al., 1999 [63]	United States of America		Cross-sectional design	*n* = 50, sex not reported, 10.6 years	68% had HbSS 28% HbSC 4% had other diagnoses (S/HPFH and Sb1)	*n* = 52 healthy children of the hospital employees, 11.0 years	WISC-III (IQ)	Compared to healthy controls, children with SCD without evidence of stroke showed subtle T1 abnormality in all grey matter structures (caudate, putamen, thalamus, cortex, and nucleus pulvinaris) examined, while no abnormalities were found in any white matter regions.Several participants with SCD showed neuropsychological impairments on psychometric measures despite no MRI abnormalities, suggesting that cognitive testing may be more sensitive to neurological injuries than MRI scanning.
Steen et al., 2003 [28]	United States of America		Cross-sectional design	*n* = 49, 45% female, 9.5 years	100% had HbSS	None	WISC-R or WISC-III (IQ)	Children with MRI abnormalities were also cognitively impaired in measures of verbal comprehension and verbal IQ.
Van der Land et al., 2015 [64]	The Netherlands		Cross-sectional design	*n* = 38, 42% female, 12.5 years	95% had HbSS 5% HbSβ° thalassaemia	None	WISC-III or WAIS-III (IQ); Beery VMI	Did find a relationship between higher volume of WM hyperintensities and lower processing speed, full-scale IQ, and verbal IQ.
Wang et al., 1998 [65]	United States of America		Cross-sectional design	*n* = 39, 53.8% female, age range of 7 to 48 months, median age of 18 months	100% had HbSS	None	BSID; VABS; MSCA	Found that 11% of the 36 asymptomatic children with SCD had CNS anomaly, and 1 had a silent stroke on MRI. Neuropsychological scores on tests were largely within normal ranges, irrespective of MRI findings. Only two of the six participants with MRI abnormalities showed signs of developmental delay (MDIs below 80) on developmental tests.
Wang et al., 2001 [12]	United States of America		Longitudinal study	*n* = 373, 47% female, 8.1 years	68% had HbSS 32% HbSC	None	WISC-R or WISC-III (IQ); WJ-III	Children with SCD and silent infarctions had lower scores for reading and maths, performance IQ, verbal IQ, coding, and full-scale IQ when compared to those with normal MRI scans, with scores continuing to decline with increasing age.
Watkins et al., 1998 [66]	United Kingdom		Cross-sectional design	*n* = 41, 41.4% female, 10.62 years	78% had HbSS 12.2% HbSC 9.8% HbSβ thalassaemia	*n* = 15 healthy siblings, 46.6% female, 9.87 years	WCST (IQ, learning and memory); WISC-Ill or WPPSI-R (IQ)	Children with evidence of stroke on MRI performed significantly worse on tests of memory, IQ, and frontal lobe functioning (Wisconsin Card Sorting Test, WCST) when compared to sibling controls and children with normal MRI scans. On the WCST and the IQ tests, children with silent infarction had scores between the children with stroke and the sibling controls and normal MRI scans groups.
**(C)**								
**Study**	**Country**		**Study Design**	**Patient Sample: Size, Sex (%), Mean Age**	**Sample Disease Characteristics**	**Control Sample**	**Neuropsychological Measures**	**Main Findings**
Chen et al., 2009 [67]	United States of America		Cross-sectional design	*n* = 31, 55% female, 9 years	100% had HbSS	None	KBIT-2	Found a relationship between intelligence quotient and GM volume in children with SCD.
Schatz & Buzan, 2006 [68]	United States of America		Cross-sectional design	*n* = 28 No lesion: (*n* = 12, 42% female, 12.3 years) Silent infarct: (*n* = 8, 25% female, 12.2 years) Overt stroke: (*n* = 8, 75% female, 13.9 years)	100% had HbSS	*n* = 16, 44% female, 13.8 years	WISC-III; the letter fluency condition of the Verbal Fluency Test; and the Self-Ordered Pointing Test (SOPT)	Found the CC was reduced for children with overt or silent infarctions compared to those without visible strokes and controls. The size of the CC was associated with working memory, speed production, and distractibility.
**(D)**								
**Study**	**Country**		**Study Design**	**Patient Sample: Size, Sex (%), Mean Age**	**Sample Disease Characteristics**	**Control Sample**	**Neuropsychological Measures**	**Main Findings**
Scantlebury et al., 2011 [69]	Canada		Retrospective study	*n* = 15, 53% female, 11.67 years	73% had HbSS 20% HbSC 7% HbSD	*n* = 10, sex not reported, 10.19 years	WISC-III or IV; WAIS-III (IQ, working memory and processing); (CPT)—II (sustained visual attention)	Found a significant relationship between core cognitive functioning (processing speed and working memory) and subtle white matter damage.
Stotesbury et al., 2018 [70]	United Kingdom		Cross-sectional design	*n* = 37, 46% female, 16.34 years median age	100% had or HbSβ° thalassaemia	*n* = 32, 57% female, 15.26 years median age	WASI-II; WISC-IV; WAIS-IV (IQ, processing speed)	Children with SCA exhibited impairments to processing speed, which was associated with damage to several white matter microstructures, including the CC and the internal capsule.
**(E)**								
**Study**	**Country**		**Study Design**	**Patient Sample: Size, Sex (%), Mean Age**	**Sample Disease Characteristics**	**Control Sample**	**Neuropsychological Measures**	**Main Findings**
Colombatti et al., 2016 [71]	Italy		Cross-sectional design	*n* = 40, 47.5% female, 8 years	98% had HbSS 2% HbSβ° thalassaemia	*n* = 16, 69% female, 9 years	WISC-III and WPSSI	Found a relationship between poor performance on cognitive tasks and greater functional connectivity in the default-mode network in patients with SCD.
Zou et al., 2011 [41]	United States of America		Cross-sectional design	*n* = 23, 35% female, 12.4 years	100% had HbSS	*n* = 21, 33% female, 12.3 years	WASI (IQ)	Found an association between performance on the verbal subtest of the WASI and BOLD signal amplitude within the visual cortex. Patients with SCA who exhibited lowered fMRI activation also had lower WASI scores.

Abbreviations: haemoglobin SS disease (HbSS), haemoglobin SC disease (HbSC), haemoglobin SB + (beta) thalassaemia (HbSβ + thalassaemia), haemoglobin SB 0 (Beta-zero) thalassaemia (HbSβ° thalassaemia), haemoglobin SD disease (HbSD), grey matter (GM), white matter (WM), corpus callosum (CC), Brigance Preschool Screen-II (BPS-II), Kaufman Brief Intelligence Test (KBIT-*2*), The Wechsler Intelligence Scale for Children—Third Edition (WISC-III) and the Wechsler Preschool and Primary Scale of Intelligence—Revised (WPPSI), The Wechsler Intelligence Scale for Children-Revised (*WISC*-*R),* Wide Range Assessment of Memory and Learning (WRAML), Beery Visual Motor Integration Test–Third Re-vision (Beery-VMI), The Wechsler Adult Intelligence Scale, Third Edition (*WAIS*-*III*), Bayleys Infant Neurodevelopmental Screener (BINS), The Trail Making Test (*TMT*), The Children’s Memory Scale (CMS), The Developmental Test of Visual–Motor Integration (VMI), The Conners’ *Continuous* Performance Test II (*CPT II*), The Tower of London (TOL), Test of Language Development–Primary, Third Edition (TOLD-P:3), Memory for Words and Decision Speed tests of the Woodcock–Johnson Tests of Cognitive Abilities, 3rd Edition (WJ-III), The *Denver Developmental Screening Test*-II (DDST-II), apparent diffusion coefficient (ADC), Woodcock–Johnson Revised, Tests of Achievement (WJ-R), Purdue Pegboard, Rapid Automatized Naming (RAN), Wisconsin Card Sorting Test (WCST), Bayley Scales of Infant Development (BSID), Vineland Adaptive Behavior Scales (VABS), McCarthy Scales of Children’s Abilities (MSCA), Children’s Auditory Verbal Learning Test (CAVLT), California Verbal Learning Test (CVLT), Test of Variables of Attention (T.O.V.A). 3.2, methodological quality of articles.

**Table 4:** Quality appraisal of studies included in the systematic review.

Study	Q1	Q2	Q3	Q4	Q5	Q6	Q7	Q8	Q9	Q10	Q11	Q12	Grade
Armstrong et al., 1996 [11]	1	1	1	0	1	1	1	1	1	1	1	1	Good
Aygun et al., 2011 [45]	1	1	1	0	1	1	1	1	0	0	1	1	Satisfactory
Bernaudin et al., 2000 [47]	1	1	1	1	1	1	1	1	1	1	1	0	Good
Brown et al., 2000 [54]	1	1	1	0	1	1	1	1	1	1	0	1	Good
Chen et al., 2009 [67]	1	1	1	0	0	1	1	1	1	1	0	1	Satisfactory
Colombatti et al., 2016 [71]	1	1	1	1	1	1	1	1	1	1	0	0	Good
Craft et al., 1993 [55]	1	1	1	1	0	1	1	1	0	1	0	0	Satisfactory
Gold et al., 2008 [56]	1	1	1	0	1	1	1	1	1	1	0	1	Good
Grueneich et al., 2004 [57]	1	1	1	1	0	1	1	1	0	1	1	1	Good
Hijmans et al., 2011 [48]	1	1	1	0	0	1	1	1	0	0	0	1	Satisfactory
Hogan et al., 2006 [43]	1	1	0	1	0	0	1	1	1	0	0	1	Satisfactory
Jacob et al., 2022 [58]	1	1	1	1	1	1	1	1	0	1	0	0	Satisfactory
Kral et al., 2006 [50]	1	1	1	0	0	0	1	1	1	1	0	1	Satisfactory
Kral et al., 2003 [49]	1	1	1	0	1	0	1	1	1	1	0	1	Satisfactory
Montanaro et al., 2013 [59]	1	1	1	0	1	1	1	1	0	0	0	1	Satisfactory
Onofri et al., 2012 [51]	1	1	1	0	0	1	1	1	0	0	0	1	Satisfactory
Prussien et al., 2019 [52]	1	1	1	0	1	1	1	1	1	0	0	1	Satisfactory
Sanchez et al., 2010 [46]	1	1	1	0	1	0	1	1	1	1	0	1	Satisfactory
Scantlebury et al., 2011 [69]	1	1	0	1	0	1	1	1	1	1	0	1	Satisfactory
Schatz & Buzan, 2006 [68]	1	1	0	1	0	1	1	1	1	1	0	1	Satisfactory
Schatz et al., 1999 [60]	1	1	1	1	0	1	1	1	1	1	0	1	Good
Schatz et al., 2008 [44]	1	1	1	0	1	1	1	1	1	0	1	1	Good
Schatz et al., 2002 [61]	1	1	0	0	0	1	1	1	1	0	0	1	Satisfactory
Steen et al., 2003 [28]	1	1	1	0	1	1	1	1	1	1	0	1	Good
Steen et al., 1998 [62]	1	1	1	1	0	1	1	1	1	1	1	1	Good
Steen et al., 1999 [63]	1	1	1	1	1	1	1	1	0	1	1	0	Good
Stotesbury et al., 2018 [70]	1	1	0	1	1	1	1	1	1	1	0	1	Good
Strouse et al., 2006 [53]	1	1	1	0	0	1	1	1	0	0	1	1	Satisfactory
van der Land et al., 2015 [64]	1	1	1	0	1	1	1	1	1	0	0	1	Satisfactory
Wang et al., 2001 [12]	1	1	1	0	1	0	1	1	1	0	1	1	Satisfactory
Wang et al., 1998 [65]	1	1	0	0	1	1	1	1	0	1	0	0	Satisfactory
Watkins et al., 1998 [66]	1	1	0	1	1	1	1	1	1	1	0	1	Good
Zou et al., 2011 [41]	1	1	0	1	0	1	1	1	1	0	0	0	Satisfactory

Score: Yes = 1, No/NA = 0, *Grade*: Poor = 0–50%, satisfactory = 51–75%, good = 76–100%.

## Data Availability

Data will be made available on direct request to the authors.
